# Study on pathogenic bacteria distribution and antimicrobial resistance in adult urinary tract infections in a tertiary hospital in Southern Jiangxi, China, 2021–2024

**DOI:** 10.3389/fcimb.2026.1831994

**Published:** 2026-07-03

**Authors:** Zhengting Liu, Liqin Zhang, Cong Liu

**Affiliations:** Department of Clinical Laboratory, Ganzhou Hospital-Nanfang Hospital, Southern Medical University (Ganzhou People's Hospital), Ganzhou, China

**Keywords:** antimicrobial resistance, carbapenem-resistant *Klebsiella pneumoniae*, epidemiological surveillance, urinary tract infection, vancomycin-resistant *Enterococcus faecium*

## Abstract

**Background:**

Urinary tract infections (UTIs) are common global bacterial infections. Rising antimicrobial resistance complicates their treatment. Analyzing pathogen distribution and resistance patterns in southern Jiangxi, China, is crucial for informing empirical treatment and infection management.

**Methods:**

We conducted a retrospective study of 3,212 urinary isolates from adult UTI patients at our hospital from January 2021 to December 2024. We used WHONET 5.6 software to analyze the distribution of pathogens and antimicrobial resistance patterns, and compared these patterns across different years, age groups, genders, and wards using the χ² test.

**Results:**

Gram-negative bacteria predominated (75.37%). *Escherichia coli* was the most prevalent at 49.00%, with *Klebsiella pneumoniae* following at 9.34%. Carbapenem-resistant *Klebsiella pneumoniae* (CRKP) increased significantly. Its resistance to imipenem rose from 4.2% in 2021 to 24.5% in 2024. Vancomycin-resistant *Enterococcus faecium* (VREfm) emerged, reaching 9.4% in 2024. CRKP resistance was highest in Intensive Care Units (ICUs) (30.8%), compared with other clinical departments.

**Conclusion:**

This study systematically analyzes the dynamic changes in pathogen profiles and antimicrobial resistance of adult urinary tract infections in southern Jiangxi from 2021 to 2024. It highlights the concurrent, rapid rise of CRKP and VREfm. The findings show a serious threat from multidrug-resistant bacteria, urging targeted infection control and antimicrobial stewardship.

## Introduction

Urinary tract infections (UTIs) are clinically common bacterial infectious diseases (Flores-Mireles et al., 2015; He et al., 2025). Globally, bacterial antimicrobial resistance (AMR) is posing an increasingly severe public health threat. Research shows that around 4.95 million global deaths in 2019 were linked to bacterial antimicrobial resistance (Antimicrobial RC, 2022). Further research specifies that, among these, drug-resistant infections attributed to urinary tract pathogens led to about 64.89 thousand deaths (Li et al., 2022). Modeling forecasts indicate that, in the absence of effective interventions, global deaths due to bacterial AMR could reach approximately 8.22 million annually by 2050 (GBD ARC, 2024).

Empirical UTI treatment is challenging due to geographical variations in pathogen composition and resistance, necessitating reliance on precise local epidemiological data. For example, data from southwestern China and Bangladesh show that the resistance rates of the predominant pathogen, *Escherichia coli*, to fluoroquinolones and third-generation cephalosporins exceed 50% (Islam et al., 2022; Zhong et al., 2025). In contrast, resistance rates to the same classes of antimicrobials are below 10% in the Germany (Naber et al., 2023). This stark contrast highlights the necessity of using region-specific data to guide clinical practice.

However, in southern Jiangxi Province, China, systematic research data on the long-term distribution of pathogens and antimicrobial resistance trends in adult UTIs has been historically lacking. This gap results in a deficiency of precise local evidence to support clinical anti-infective practices in this region. Therefore, this retrospective study examined pathogen distribution and antimicrobial resistance trends in adult UTI patients at a major tertiary hospital in southern Jiangxi from 2021 to 2024. The study seeks to clarify regional epidemiological traits and supply essential data to optimize empirical anti-infective treatments, implement precise antimicrobial stewardship, and improve infection control strategies in the area.

## Materials and methods

### Study design

This retrospective observational study was conducted at Ganzhou People’s Hospital in Jiangxi Province. Clinical microbiology data from January 1, 2021, to December 31, 2024, were analyzed. The hospital’s Ethics Review Committee approved the study protocol (Approval No: PJB2025-165-01). The ethics committee waived the need for written informed consent due to the study’s retrospective design. The process followed the ethical guidelines outlined in the Declaration of Helsinki. To prevent duplicate counting, the analysis included only the initial isolate of each pathogen per patient.

### Data sampling and processing procedure

To construct the analysis dataset, all urine culture reports within the study period were exported from the Laboratory Information System (LIS). These reports included patient identification codes, specimen collection dates, microbiological results (identified pathogens), and corresponding antimicrobial susceptibility testing data. The exported data were then directly imported into WHONET 5.6 software for management and analysis. For the overall analysis covering the entire 2021–2024 period, we retained only the first positive urine culture per patient across the whole 4-year study period to avoid overrepresentation of individuals with recurrent infections. For the annual trend analysis, we retained the first positive culture per patient within each calendar year (i.e., a patient could contribute one isolate per year if they had infections in different years). To maintain data quality and prevent duplicate counts within each analytical approach, WHONET 5.6 software was used to filter multiple positive urine cultures accordingly.

### Sample collection

**Figure 1:**
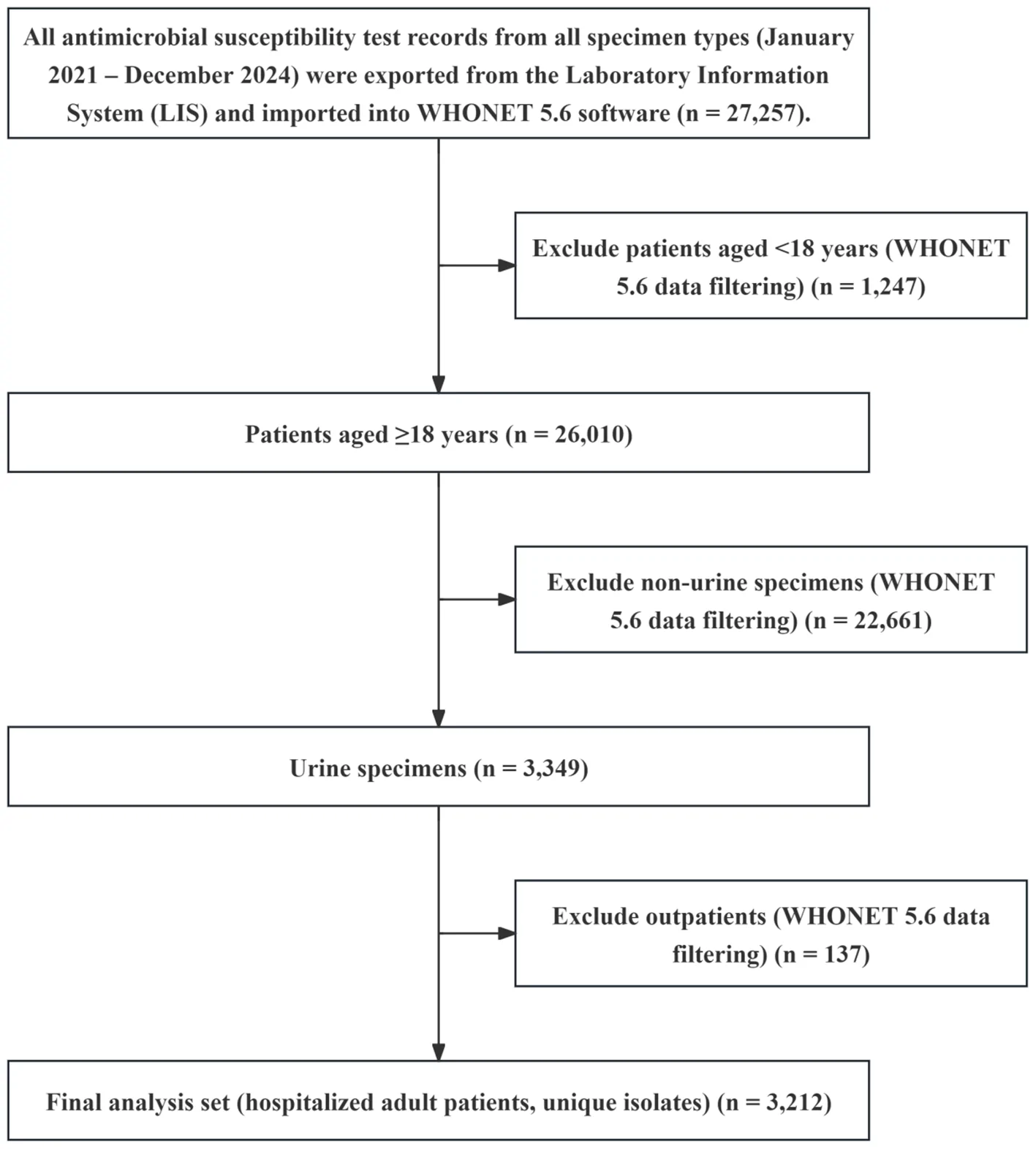
Flowchart of patient inclusion and exclusion for urinary tract infection.

The study participants were hospitalized patients clinically diagnosed with a urinary tract infection and aged ≥18 years. The exclusion criteria were (1): age <18 years (2); colony counts below the positive reporting threshold (<10^4^ CFU/mL for Gram-positive cocci, <10^5^ CFU/mL for Gram-negative bacilli) (3); contaminated specimens (growth of ≥3 distinct microorganisms) (4); outpatients (5); duplicate isolates from the same patient (only the first isolate of each species per patient was retained). The detailed inclusion and exclusion process is illustrated in **Figure 1**.

### Definition of urinary tract infection

In this study, urinary tract infection (UTI) was defined as meeting all three of the following criteria: (1) clinical symptoms: presence of at least one urinary-specific symptom (dysuria, frequency, urgency, suprapubic pain, or fever >38.0 °C); (2) urinalysis indicating infection: positive dipstick for leukocyte esterase or nitrite; (3) positive urine culture: clean-catch midstream urine with colony counts ≥10^5^ CFU/mL for Gram-negative bacilli or ≥10^4^ CFU/mL for Gram-positive cocci.

### Definition of carbapenem-resistant enterobacterales

According to the Clinical and Laboratory Standards Institute (CLSI) M100 document, 34th edition (CLSI M100-Ed34:2024) breakpoints (imipenem MIC ≥4 μg/mL; ertapenem MIC ≥2 μg/mL), isolates resistant to either imipenem or ertapenem are considered carbapenem-resistant. For organisms with intrinsically reduced susceptibility to imipenem (*e.g., Morganella spp., Proteus spp.,* and *Providencia spp.*), susceptibility results for other carbapenems (e.g., ertapenem) should be used for interpretation.

### Urine culture and pathogen identification

Clean-catch midstream urine samples were inoculated onto 5% sheep blood agar and China Blue agar plates (Detgerm, China) within 2 hours of collection. A 10 μL inoculating loop was used for streaking, and the samples were incubated aerobically at 35 ± 2 °C for 18–24 hours. Bacterial colonies of significant growth were selected for further identification. Species identification was conducted using the VITEK MS system (bioMérieux, France) for MALDI-TOF MS, following the manufacturer’s standard protocol.

### Antibiotic susceptibility testing

Antimicrobial susceptibility testing (AST) was routinely performed following a standardized laboratory protocol. The VITEK^®^ 2 Compact automated system, utilizing specialized AST cards from bioMérieux, France, was used to primarily determine the minimum inhibitory concentration. Inoculum preparation and loading were performed in strict accordance with the manufacturer’s guidelines. For specific antibiotics not on the AST card or to verify anomalous/indeterminate results from the automated system, the conventional Kirby-Bauer disk diffusion method (using disks from Kangtai, China) was employed as a supplementary or confirmatory test. AST results, including MIC and zone diameters, were interpreted using the breakpoints from the CLSI M100–34 guidelines. Quality control was essential to the routine protocol, involving concurrent testing of standard reference strains (*Staphylococcus aureus* ATCC 25923, ATCC 29213; *Escherichia coli* ATCC 25922; *Pseudomonas aeruginosa* ATCC 27853) with each batch of clinical isolates. Only data from batches where control strains yielded results within the acceptable ranges were validated and included in the final analysis.

### Statistical analysis

WHONET 5.6 software was used for the management and descriptive statistical analysis of the antimicrobial susceptibility data. Graphs and charts were generated using OriginPro 2023 software (OriginLab, USA). Categorical variables are shown as frequencies (percentages), with group comparisons conducted via Pearson’s chi-square test or Fisher-Freeman-Halton exact test. Statistical significance was defined as a P-value less than 0.05.

## Results

### Study population and baseline characteristics

A total of 3,212 unique patients with their first positive urine culture during the entire 4-year study period (2021–2024) were included in the overall analysis. For annual trend analysis, we included the first positive culture per patient within each calendar year, resulting in 839 isolates in 2021, 710 in 2022, 766 in 2023, and 982 in 2024. **Table 1** presents the demographic details of the patients. Among the 3,212 isolates, 1,956 (60.9%) were derived from female patients and 1,256 (39.1%) from male patients. The distribution of strains by age group was as follows: 460 strains (14.32%) from patients aged 18–44 years, 1,341 strains (41.75%) from those aged 45–64 years, and 1,411 strains (43.93%) from patients aged 65 years and older. The majority of isolates originated from patients in general wards (2,961 [92.19%]), whereas 251 (7.81%) were from ICU patients.

**Table 1:** Baseline characteristics of the patients.

Variables	Number of patients (n)	Percentage (%)
Gender		
Male	1,256	39.1
Female	1,956	60.9
Age (years)		
18-44	460	14.32
45-64	1,341	41.75
≥65	1,411	43.93
Ward		
ICU	251	7.81
General ward	2,961	92.19
Total	3,212	100

### Pathogen distribution

A total of 3,212 patients with urinary tract infection were enrolled. **Table 2** indicates that Gram-negative bacteria constituted 75.37% (2,421/3,212) of the total, with *Escherichia coli* as the predominant species (49.00%), followed by *Klebsiella pneumoniae* (9.34%) and *Proteus mirabilis* (4.02%). Gram-positive bacteria comprised 19.46%, predominantly *Enterococcus faecalis* (7.44%) and *Enterococcus faecium* (6.85%). Fungi accounted for 5.17%, with *Candida albicans* being the most frequent (2.18%).

**Table 2:** Distribution characteristics of uropathogens in urinary tract infections.

Variables	Number of strains (n)	Percentage (%)
Gram-negatives bacteria	2,421	75.37
*Escherichia coli*	1,574	49.00
*Klebsiella pneumoniae*	300	9.34
*Proteus mirabilis*	129	4.02
*Pseudomonas aeruginosa*	86	2.68
*Enterobacter cloacae*	74	2.30
Others	258	8.03
Gram-positives bacteria	625	19.46
*Enterococcus faecalis*	239	*7.44*
*Enterococcus faecium*	220	*6.85*
*Staphylococcus aureus*	60	*1.87*
*Streptococcus agalactiae*	55	*1.71*
Others	51	1.59
Fungi	166	5.17
*Candida albicans*	70	2.18
*Candida glabrata*	46	1.43
*Candida tropicalis*	34	1.06
Others	16	0.50
Total	3,212	100

**Supplementary Figure 1** illustrates the main differences observed in subgroup analysis. Regarding gender, the proportion of *Escherichia coli* was significantly higher in female patients (60.0%) than in male patients (33%), whereas male patients had higher proportions of *Enterococcus faecalis* (12% vs 5%) and *Enterococcus faecium* (8% vs 6%). With respect to age, the proportion of *Escherichia coli* was lower in the ≥65 years group (44%) compared with the 18–44 years (51%) and 45–64 years (53%) groups; the proportion of *Enterococcus faecium* increased with age (5% in 45–64 years, 9% in ≥65 years), while *Enterococcus faecalis* was highest in the 18–44 years group (10%).

Department-stratified analysis (**Supplementary Figure 2**) showed that *Escherichia coli* was the most common uropathogen in all departments, accounting for 36%–73% of isolates, with the highest proportion in Gynecology (73%) and the lowest in the Rehabilitation Medicine (36%). *Enterococcus faecium* had relatively higher proportions in the ICU (17%), General Practice (13%), and Emergency Medicine (11%), while *Enterococcus faecalis* was more common in the Urology (10%), Nephrology (7%), and Oncology (7%). *Klebsiella pneumoniae* accounted for 5%–22% of isolates across departments. *Candida albicans* was detected in the ICU (6%) and Emergency Medicine (4%).

### Antimicrobial resistance of major gram-negative bacteria

**Table 3** illustrates the variations in resistance rates of *Escherichia coli*, *Klebsiella pneumoniae*, and *Proteus mirabilis* to frequently used antimicrobial agents from 2021 to 2024. In *Escherichia coli*, significant annual variations were found in resistance rates to ertapenem (0.8%–3.7%, *p* = 0.027) and imipenem (0.5%–2.8%, *p* = 0.050), while resistance rates to other beta-lactams, aminoglycosides, fluoroquinolones, and sulfonamides remained statistically unchanged (*p* > 0.05). For *Klebsiella pneumoniae*, resistance rates varied significantly over the years for cefoperazone-sulbactam (7.0%–29.5%, *p* < 0.001), ertapenem (5.1%–24.5%, *p* < 0.001), and imipenem (3.8%–23.2%, *p* < 0.001). For *Proteus mirabilis*, the resistance rate to imipenem varied significantly over the study period (4.5% to 43.8%, *p* < 0.001), while rates for other antimicrobial agents showed no significant annual changes.

**Table 3:** Changes in antimicrobial susceptibility of major gram-negative rods causing urinary tract infections over time.

Antibiotics	*Escherichia coli*								*p*
	2021 (n = 408)		2022 (n = 346)		2023 (n = 385)		2024 (n = 492)		
	Resistant	Non-resistant	Resistant	Non-resistant	Resistant	Non-resistant	Resistant	Non-resistant	
ESBL	49.4	50.6	48.2	51.8	49.7	50.3	48.8	51.2	0.980
AMC	12.2	87.8	9.6	90.4	9.8	90.2	11.5	88.5	0.554
CSL	4.7	95.3	6.4	93.6	4.9	95.1	5.3	94.7	0.750
TZP	4.7	95.3	5.8	94.2	6.2	93.8	6.1	93.9	0.758
CXM	54.1	45.9	54	46	53.8	46.2	52.8	47.2	0.978
CAZ	23.2	76.8	21.6	78.4	24.5	75.5	24.4	75.6	0.791
CRO	51.1	48.9	52	48	51.9	48.1	53	47	0.943
FEP	11.4	88.6	11.9	88.1	10.4	89.6	11.3	88.7	0.931
FOX	13.9	86.1	10.5	89.5	10.3	89.7	13	87	0.293
ETP	1.7	98.3	2	98	0.8	99.2	3.7	96.3	0.027
IPM	1.2	98.8	1.7	98.3	0.5	99.5	2.8	97.2	0.050
AMK	2	98	3.2	96.8	3.1	96.9	2.9	97.1	0.707
LEV	51.8	48.2	51.7	48.3	54	46	57.3	42.7	0.290
SXT	51.1	48.9	47.1	52.9	45.1	54.9	50.2	49.8	0.317
Antibiotics	*Klebsiella pneumoniae*								*p*
	2021 (n = 71)		2022 (n = 79)		2023 (n = 64)		2024 (n = 95)		
	Resistant	Non-resistant	Resistant	Non-resistant	Resistant	Non-resistant	Resistant	Non-resistant	
ESBL	45.7	54.3	39.2	60.8	41.3	58.7	28.7	71.3	0.142
AMC	15.5	84.5	22.8	77.2	23.8	76.2	34.4	65.6	0.036
CSL	7	93	11.4	88.6	9.4	90.6	29.5	70.5	<0.001
TZP	15.5	84.5	15.6	84.4	22.2	77.8	29.8	70.2	0.071
CXM	56.3	43.7	46.8	53.2	50	50	55.8	44.2	0.571
CAZ	36.6	63.4	35.4	64.6	34.9	65.1	39.4	60.6	0.939
CRO	53.5	46.5	44.3	55.7	45.3	54.7	52.6	47.4	0.547
FEP	35.2	64.8	37.7	62.3	31.7	68.3	43.6	56.4	0.470
FOX	16.9	83.1	13.9	86.1	17.5	82.5	28.7	71.3	0.078
ETP	5.6	94.4	5.1	94.9	7.9	92.1	24.5	75.5	<0.001
IPM	4.2	95.8	3.8	96.2	6.2	93.8	23.2	76.8	<0.001
AMK	11.3	88.7	6.3	93.7	9.4	90.6	18.9	81.1	0.065
LEV	39.4	60.6	39.2	60.8	35.9	64.1	40	60	0.961
SXT	50.7	49.3	46.8	53.2	39.1	60.9	42.1	57.9	0.277
Antibiotics	*Proteus mirabilis*								*p*
	2021 (n = 28)		2022 (n = 33)		2023 (n = 28)		2024 (n = 44)		
	Resistant	Non-resistant	Resistant	Non-resistant	Resistant	Non-resistant	Resistant	Non-resistant	
AMC	25	75	18.2	81.8	44.4	55.6	22.7	77.3	0.145
CSL	0	100	0	100	0	100	0	100	
TZP	0	100	0	100	0	100	0	100	
CXM	35.7	64.3	33.3	66.7	53.6	46.4	43.2	56.8	0.388
CAZ	0	100	6.1	93.9	0	100	2.3	97.7	0.256
CRO	32.1	67.9	27.3	72.7	39.3	60.7	36.4	63.6	0.761
FEP	0	100	0	100	0	100	0	100	
FOX	0	100	3	97	7.4	92.6	9.1	90.9	0.198
ETP	0	100	0	100	0	100	0	100	
IPM	4.5	95.5	43.8	56.2	39.3	60.7	26.8	73.2	<0.001
AMK	3.6	96.4	0	100	0	100	0	100	0.370
LEV	35.7	64.3	33.3	66.7	53.6	46.4	31.8	68.2	0.265
SXT	64.3	35.7	57.6	42.4	64.3	35.7	68.2	31.8	0.819

AMC, amoxicillin–clavulanate; CSL, cefoperazone–sulbactam; TZP, piperacillin–tazobactam; CXM, cefuroxime; CAZ, ceftazidime; CRO, ceftriaxone; FEP, cefepime; FOX, cefoxitin; ETP, ertapenem; IPM, imipenem; AMK, amikacin; LEV, levofloxacin; SXT, trimet hoprim–sulfamethoxazole; ESBL, Extended-Spectrum Beta-Lactamase.

### Antimicrobial resistance of major gram-positive bacteria

**Table 4** presents the resistance rate variations of *Enterococcus faecium* and *Enterococcus faecalis* from 2021 to 2024. *Enterococcus faecium* exhibited resistance rates exceeding 95% for penicillin, ampicillin, and levofloxacin consistently over the years, with no notable variations. The rate of high-level gentamicin resistance escalated from 15.4% to 57.1% (*p* < 0.001), while vancomycin resistance increased from 0% to 9.4% (*p* = 0.020). *Enterococcus faecalis* exhibited low resistance rates to penicillin, ampicillin, and vancomycin, with no significant changes observed in high-level gentamicin, levofloxacin, and linezolid resistance over the study period.

**Table 4:** Changes in antimicrobial susceptibility of major gram-positive cocci causing urinary tract infections over time.

Antibiotics	*Enterococcus faecium*								*p*
	2021 (n = 65)		2022 (n =40)		2023 (n = 54)		2024 (n = 64)		
	Resistant	Non-resistant	Resistant	Non-resistant	Resistant	Non-resistant	Resistant	Non-resistant	
PEN	100	0	100	0	100	0	98.4	1.6	0.474
AMP	98.5	1.5	100	0	96.3	3.7	98.4	1.6	0.503
GEH	15.4	83.8	33.3	66.7	32.1	67.9	57.1	42.9	<0.001
LEV	96.9	2.9	100	0	100	0	96.9	3.1	0.218
LNZ	1.5	100	0	100	0	100	1.6	98.4	0.531
VAN	0	100	2.5	97.5	1.9	98.1	9.4	90.6	0.020
Antibiotics	*Enterococcus faecalis*								*p*
	2021 (n =70)		2022 (n = 51)		2023 (n = 55)		2024 (n = 67)		
	Resistant	Non-resistant	Resistant	Non-resistant	Resistant	Non-resistant	Resistant	Non-resistant	
PEN	1.4	98.6	2	98	0	100	1.5	98.5	0.657
AMP	0	100	0	100	0	100	0	100	
GEH	29.9	70.1	29.8	70.2	42.6	57.4	34.8	65.2	0.479
LEV	24.3	75.7	33.3	66.7	23.6	76.4	34.3	65.7	0.404
LNZ	8.6	91.4	4	96	1.8	98.2	3.1	96.9	0.286
VAN	0	100	0	100	0	100	0	100	

PEN, penicillin; AMP, ampicillin; GEH, high-level gentamicin; LNZ, linezolid; VAN, vancomycin; LEV, levofloxacin.

### Changes in resistance rates of multidrug-resistant bacteria

**Figure 2:**
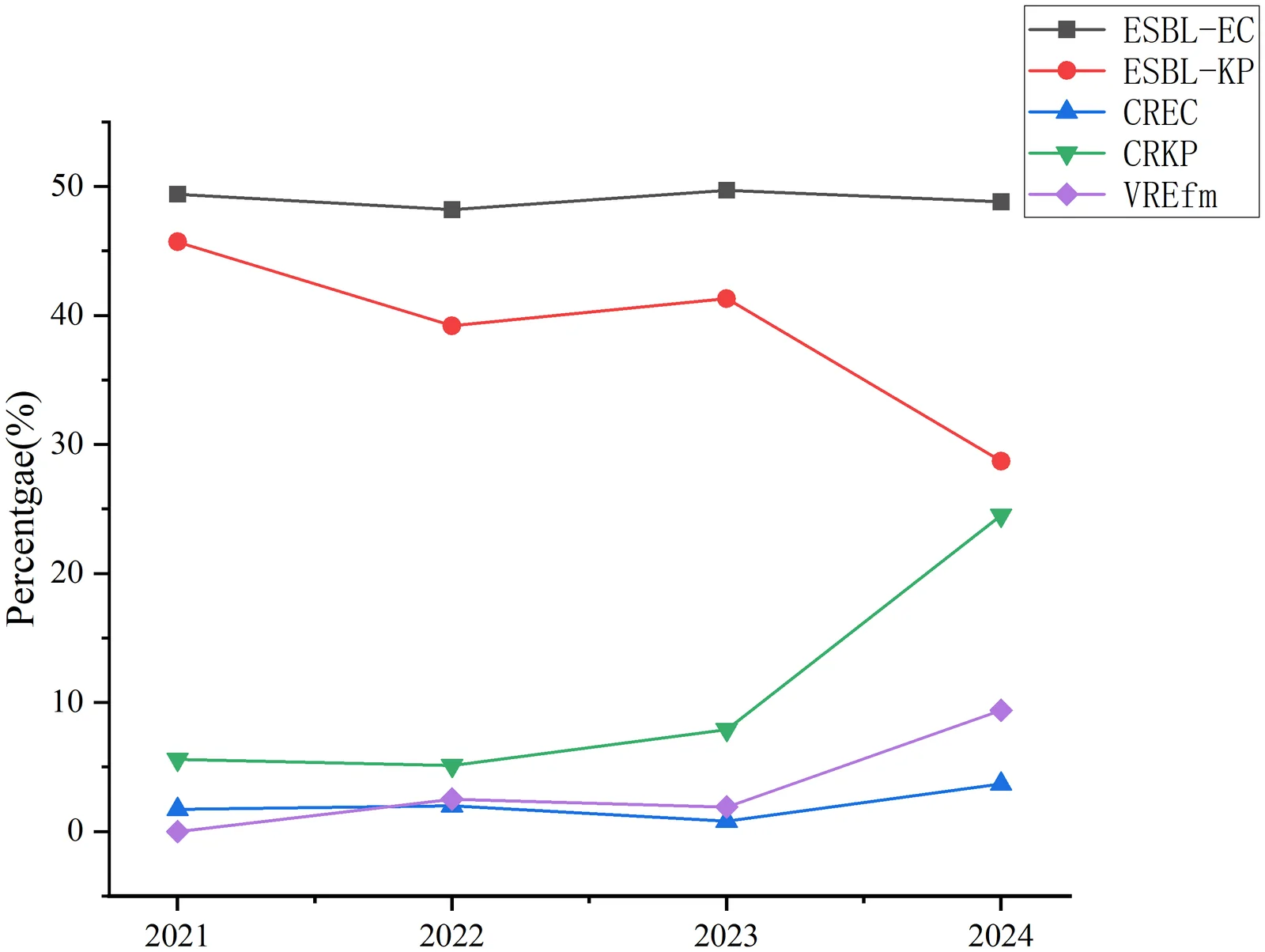
Annual prevalence of major antimicrobial-resistant pathogens, 2021–2024. CREC, Carbapenem-Resistant *Escherichia coli*; CRKP, Carbapenem-Resistant *Klebsiella pneumoniae*; VREfm, Vancomycin-Resistant *Enterococcus faecium*; ESBL-EC, ESBL-producing *Escherichia coli*; ESBL-KP, ESBL-producing *Klebsiella pneumoniae*.

**Figure 2** shows the annual prevalence of major antimicrobial-resistant pathogens from 2021 to 2024. The detection rate of ESBL-producing *Escherichia coli* (ESBL-EC) remained stable, decreased from 49.4% to 48.8%. ESBL-producing *Klebsiella pneumoniae* (ESBL-KP) decreased from 45.7% in 2021 to 28.7% in 2024. Carbapenem-resistant *Escherichia coli* (CREC) fluctuated between 1.2% and 3.7% without a clear trend. In contrast, CRKP rose markedly from 4.2% in 2021 to 24.5% in 2024. VREfm increased from 0% in 2021 to 9.4% in 2024.

### Antimicrobial resistance analysis based on population stratification

#### Gram-negative bacteria

**Supplementary Table 1** compares the resistance rates of major Gram-negative bacteria across different patient subgroups. Gender-based differences were the most prominent feature, with consistently higher resistance rates in male than in female patients for both *Escherichia coli* (e.g., ceftriaxone 60.6% vs 48.8%, ertapenem 3.8% vs 1.6%; all *p* < 0.05) and *Klebsiella pneumoniae* (e.g., imipenem 17.9% vs 5.9%; *p* < 0.05). Age-related differences were not consistent across organisms, and for *Proteus mirabilis*, only cefuroxime resistance showed a significant gender difference (56.4% vs 33.3%, *p* = 0.014) due to the small sample size.

#### Gram-positive cocci

**Supplementary Table 2** presents the resistance rates of *Enterococcus faecalis* and *Enterococcus faecium* among different patient subgroups. *Enterococcus faecalis* was susceptible to ampicillin and vancomycin across all subgroups, but penicillin resistance was higher in males than in females (2.1% vs 0%, *p* = 0.084). No notable differences were found for the remaining antibiotics. For *Enterococcus faecium*, resistance to penicillin, ampicillin, and levofloxacin was uniformly high (89.5%–100%), with no significant differences across subgroups for any antibiotic.

### Department-stratified antimicrobial resistance rates of major uropathogens

Department-stratified analysis (**Supplementary Tables 3**, **4**) showed that among the 10 clinical departments, the resistance rate of *Escherichia coli* to ceftriaxone exceeded 50% in all departments except Nephrology, Endocrinology, and General Practice, while the carbapenem resistance rate of *Escherichia coli* remained low (<6%) in all departments. For *Klebsiella pneumoniae*, the carbapenem resistance rate was 0% in Nephrology, Endocrinology, and Oncology, but was significantly higher in the Emergency Department (18.2%), Rehabilitation Department (28.1%), Neurology (27.3%), and ICU (30.8%). *Enterococcus faecalis* remained susceptible to vancomycin, whereas *Enterococcus faecium* showed high-level resistance (close to 100% to penicillin, ampicillin, and levofloxacin), with vancomycin resistance rates of 8.7% in the Emergency Department and 2.4% in the ICU.

## Discussion

This four-year study conducted in southern Jiangxi indicates that Gram-negative bacteria are the predominant pathogens in local UTIs, which is consistent with existing literature (Li et al., 2022; Zhong et al., 2025). Notably, however, significant variations in pathogen profiles exist across different geographic regions and populations. The study identified *Escherichia coli*, *Enterococcus* spp., and *Klebsiella pneumoniae* as the most commonly isolated pathogens. In contrast, Joel Bazira et al. reported that *Escherichia coli*, *Klebsiella pneumoniae*, and *Citrobacter* spp. predominated in the Mbarara Region of Uganda (Bazira et al., 2025), while Mohammad Aminul Islam et al. found that *Escherichia coli*, *Streptococcus* spp., and *Klebsiella* spp. were the most common isolates in Dhaka, Bangladesh (Islam et al., 2022). This comparison further highlights the geographic heterogeneity of uropathogen profiles, suggesting that clinical practice should rely on local surveillance data to formulate precise diagnostic and therapeutic strategies.

Subgroup analysis further indicates that the distribution of UTIs pathogens varies by factors such as age, gender, and clinical setting. The pathogen profile varies by gender, with *Escherichia coli* being more prevalent in female UTIs (60%) compared to males (33%). Additionally, the detection rate of this bacterium rises with age, aligning with findings by Zhi-Song Zhan et al (Zhan et al., 2024). In contrast, the proportion of *Enterococcus faecalis* is significantly higher in male patients (12%) than in females (5%), a result consistent with existing literature and likely attributable to differences in anatomical and physiological characteristics (Magliano et al., 2012; Zhan et al., 2024). Additionally, higher detection rates of *Enterococcus* spp., *Candida albicans* was noted in ICU patients, likely linked to invasive procedures, immunocompromised conditions, and broad-spectrum antibiotic use (Chang et al., 2023).

This study found that the resistance rates of *Escherichia coli* to second- and third-generation cephalosporins, levofloxacin, and trimethoprim-sulfamethoxazole consistently exceeded 50%, which is consistent with the CHINET 2024 surveillance data (ceftriaxone resistance rate approximately 52%) and findings from other regions in China (Chang et al., 2023; Qin et al., 2024; Guo et al., 2025). An Italian study of over 3,000 *Escherichia coli* isolates from UTIs reported significantly lower resistance rates to third-generation cephalosporins (17%) and trimethoprim-sulfamethoxazole (24%), along with a reduced proportion of extended-spectrum β-lactamase-producing strains (12%) (Milano et al., 2022), compared to this study. These drugs are unsuitable for empirical therapy in this region, consistent with international guidelines that advise against using third-generation cephalosporins and fluoroquinolones in areas with high ESBL producer prevalence (Tamma et al., 2024). In contrast, carbapenems maintained low resistance rates (<4%) throughout the study period, consistent with reports from other regions in China (Li et al., 2024) and lower than those reported in the Mbarara area (Bazira et al., 2025), indicating their potential as a reliable therapeutic option for complicated or severe urinary tract infections in this region.

In contrast, the burden of CRKP increased significantly, with a notably concerning rapid upward trend in its resistance rate to carbapenem antibiotics, which serve as critical last-line therapeutic agents. This finding suggests that the local region has become a high-burden area for CRKP, contrasting with the lower prevalence levels reported in some European countries (Girmenia et al., 2016). The CRKP detection rate in this study was higher than recent findings from southwestern China but lower than the notably high rates in Chongming District, Shanghai, highlighting significant regional variations in bacterial resistance across China (Li et al., 2022; Zhong et al., 2025). To further contextualize our findings at the national level, we compared our data with two large-scale CHINET surveillance datasets. Compared with the CHINET urine-specific analysis (2015–2021; CRKP 20.5%) (Li et al., 2024), the CRKP rate in our region (24.5% in 2024) was higher. Compared with the CHINET 2024 hospital-wide data (CRKP 22.1%) (Guo et al., 2025), the resistance rates in our Emergency Department (18.2%), Rehabilitation Department (28.1%), and ICU (30.8%) exceeded the national average in certain high-risk departments. These comparisons indicate that the CRKP burden in southern Jiangxi, particularly in high-risk departments, is more severe than the national level. This resistance is likely mediated primarily by the production of carbapenemases, such as KPC, NDM, and OXA-48-like enzymes (Simner et al., 2024). These cross-regional variations may be attributable to the combined influence of multiple factors, including but not limited to: regional differences in antibiotic prescribing practices and usage intensity, the implementation intensity of infection prevention and control measures in healthcare settings, population mobility and epidemiological background, as well as locally prevalent resistant genotypes and clonal lineages. Furthermore, the concentration of CRKP in ICU, Emergency, Rehabilitation, and Neurology departments reinforces that intensive care units and other high-risk departments serve as epicenters for carbapenem-resistant organism transmission, driven by high antibiotic selection pressure, invasive procedures, and patient acuity (Tian et al., 2019; Bizuayehu et al., 2022).

*Proteus mirabilis* frequently causes UTIs in this area. Research indicates that infections from this organism are more clinically severe than those from *Escherichia coli*, often leading to urinary tract stone formation and a higher incidence of pyelonephritis (Meumann et al., 2015; Mo et al., 2022). In this study, this organism maintained high susceptibility rates to multiple antimicrobial agents, including compound preparations containing enzyme inhibitors (55.6%–100%), ceftazidime (97.7%), cefepime (100%), cefoxitin (90.9%), and ertapenem (100%), consistent with most domestic reports (Danilo De Oliveira et al., 2021; Mo et al., 2022; Zhong et al., 2025). It should be noted that although the susceptibility test for imipenem showed a relatively high resistance rate (26.8%), according to CLSI guidelines, *Proteus mirabilis* has a high intrinsic MIC breakpoint for imipenem. Therefore, routine susceptibility results should not be directly interpreted as carbapenem resistance and require comprehensive assessment combined with other carbapenems such as ertapenem and meropenem. Additionally, The American Medical Association recommend fluoroquinolones as the primary treatment for complicated pyelonephritis (Lee et al., 2021). However, this study showed a quinolone resistance rate of up to 30%, suggesting that it is not suitable for empirical treatment of UTIs in this region.

Simultaneously, the emergence of VREfm, from none to a concerning level, marks a critical development. Vancomycin is a cornerstone for treating severe enterococcal infections, and its failure limits therapeutic options and facilitates transmission. This study found that VREfm predominantly affected patients aged 45 years and older and was detected in the Emergency Department (8.7%) and the ICU (2.4%), providing a clear target for precision infection control, such as active screening upon admission in high-risk units. The simultaneous rise of CRKP and VREfm severely constrains empirical therapy, potentially forcing overreliance on last-resort agents like tigecycline, polymyxin, and linezolid, which in turn risks selecting for resistance to these final-line drugs.

These findings carry urgent clinical implications. The high resistance rates of *Escherichia coli* and *Klebsiella pneumoniae* to third-generation cephalosporins and fluoroquinolones (approaching or exceeding 50%) render these agents unsuitable as empirical options in this region. The concurrent presence of CRKP and VREfm may lead to clinical over-reliance on last-resort agents such as tigecycline, polymyxins, or linezolid, thereby accelerating the selection of resistance to these drugs. Consequently, a tiered strategy is imperative: empirical regimens for ICU and high-risk patients must cover these multidrug-resistant bacteria. Simultaneously, it is essential to curb their transmission by strengthening antimicrobial stewardship—enforcing strict control over the use of carbapenems and glycopeptides—and enhancing infection prevention and control measures, including active screening, contact precautions, and environmental cleaning.

This study has several limitations. First, its single-center design limits the generalizability of the findings. Second, the lack of recorded symptom onset time prevented differentiation between community-acquired and hospital-acquired infections. Third, in the department-stratified analysis, some departments (e.g., Cardiology, Geriatrics) had fewer than 30 isolates for certain pathogens and were therefore not analyzed for resistance rates, which may have resulted in missing resistance data for these departments. Moreover, multivariate regression analysis was not performed to adjust for potential confounders (e.g., comorbidities, prior antibiotic use) when comparing resistance rates across gender and age groups. Fourth, molecular data such as carbapenemase genotyping and van gene analysis were unavailable. Future multi-center prospective studies and strengthened regional surveillance networks are needed.

## Conclusion

This study indicates that the increasing prevalence of CRKP and VREfm represents the most significant challenge in treating urinary tract infections within this region. To address this, two core measures must be implemented immediately: strengthening infection control in high-risk areas and populations and implementing a tiered antimicrobial stewardship strategy based on local data. These actions are critical for improving patient outcomes and countering public health threats.

## Data Availability

The original contributions presented in the study are included in the article/**Supplementary Material**. Further inquiries can be directed to the corresponding author.
