# Correction: Study on pathogenic bacteria distribution and antimicrobial resistance in adult urinary tract infections in a tertiary hospital in Southern Jiangxi, China, 2021–2024

**DOI:** 10.3389/fcimb.2026.1955206

**Published:** 2026-09-02

**Authors:** Zhengting Liu, Liqin Zhang, Cong Liu

**Affiliations:** Department of Clinical Laboratory, Ganzhou Hospital-Nanfang Hospital, Southern Medical University (Ganzhou People’s Hospital), Ganzhou, China

**Keywords:** antimicrobial resistance, carbapenem-resistant *Klebsiella pneumoniae*, epidemiological surveillance, urinary tract infection, vancomycin-resistant *Enterococcus faecium*

The grant number “(No. 2026E01073)” for funder “Medical Leading Discipline of Ganzhou City” to “Zhengting Liu, Liqin Zhang and Cong Liu” was erroneously included. The correct **Funding** statement reads:

“The author(s) declared that financial support was received for this work and/or its publication. This work was supported by the funds of the Medical Leading Discipline of Ganzhou City (no grant number). The funder provided financial support for the research and had no role in study design, data collection, data analysis, interpretation of results, or writing of the manuscript.”

**I**n the **Results** section, under the subheading “*Pathogen distribution*,” the sentence “The lowest in the ICU (39%) should be corrected to the lowest in the Rehabilitation Medicine (36%)”. This sentence is incorrect; it should follow the preceding sentence as: “with the highest proportion in Gynecology (73%) and the lowest in the Rehabilitation Medicine (36%).”

In the **Materials and methods** section, under the subheading “*Data sampling and processing procedure*,” the last sentence “The detailed inclusion and exclusion process is illustrated in **Figure 1**” should be deleted.

In the **Materials and methods** section, under subheading “*Statistical Analysis*” ‘OriginPro 2023 software (Electronic Arts, USA)’ should be changed to ‘OriginPro 2023 software (OriginLab, USA)’. The sentence now reads:

“Graphs and charts were generated using OriginPro 2023 software (OriginLab, USA).”

The original version of this article has been updated.

