# Supplementary material for: Study on pathogenic bacteria distribution and antimicrobial resistance in adult urinary tract infections in a tertiary hospital in Southern Jiangxi, China, 2021–2024
Source: Front Cell Infect Microbiol. 2026 Jul 3;16:1831994. doi: 10.3389/fcimb.2026.1831994 (PMC13375497; doi:10.3389/fcimb.2026.1831994)
Supplement: Supplementary file 1 [file SupplementaryFile1.docx]

**Supplementary Tables**


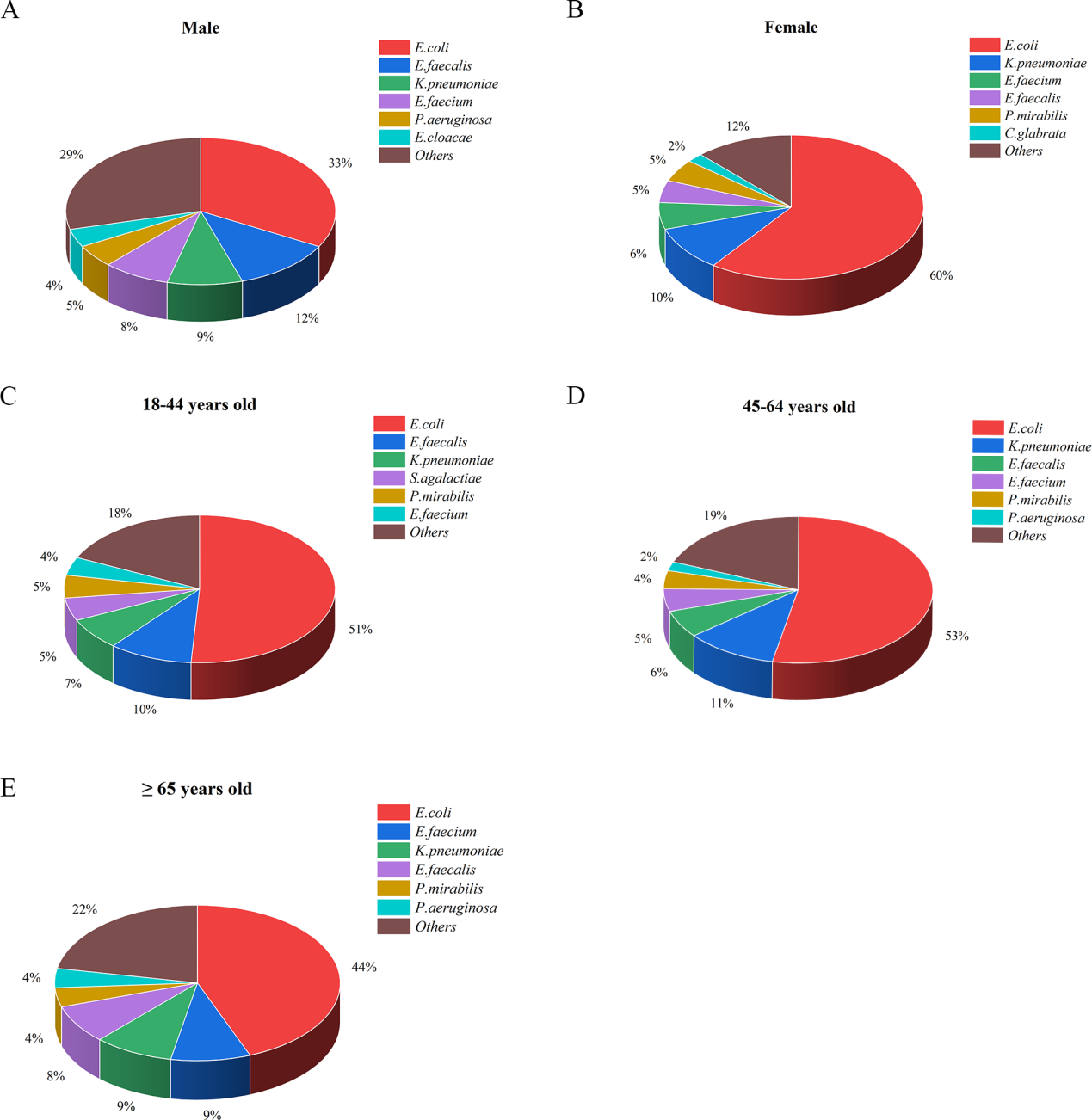


Figure S1 Distribution of uropathogens across age groups and gender among patients with urinary tract infection.

**
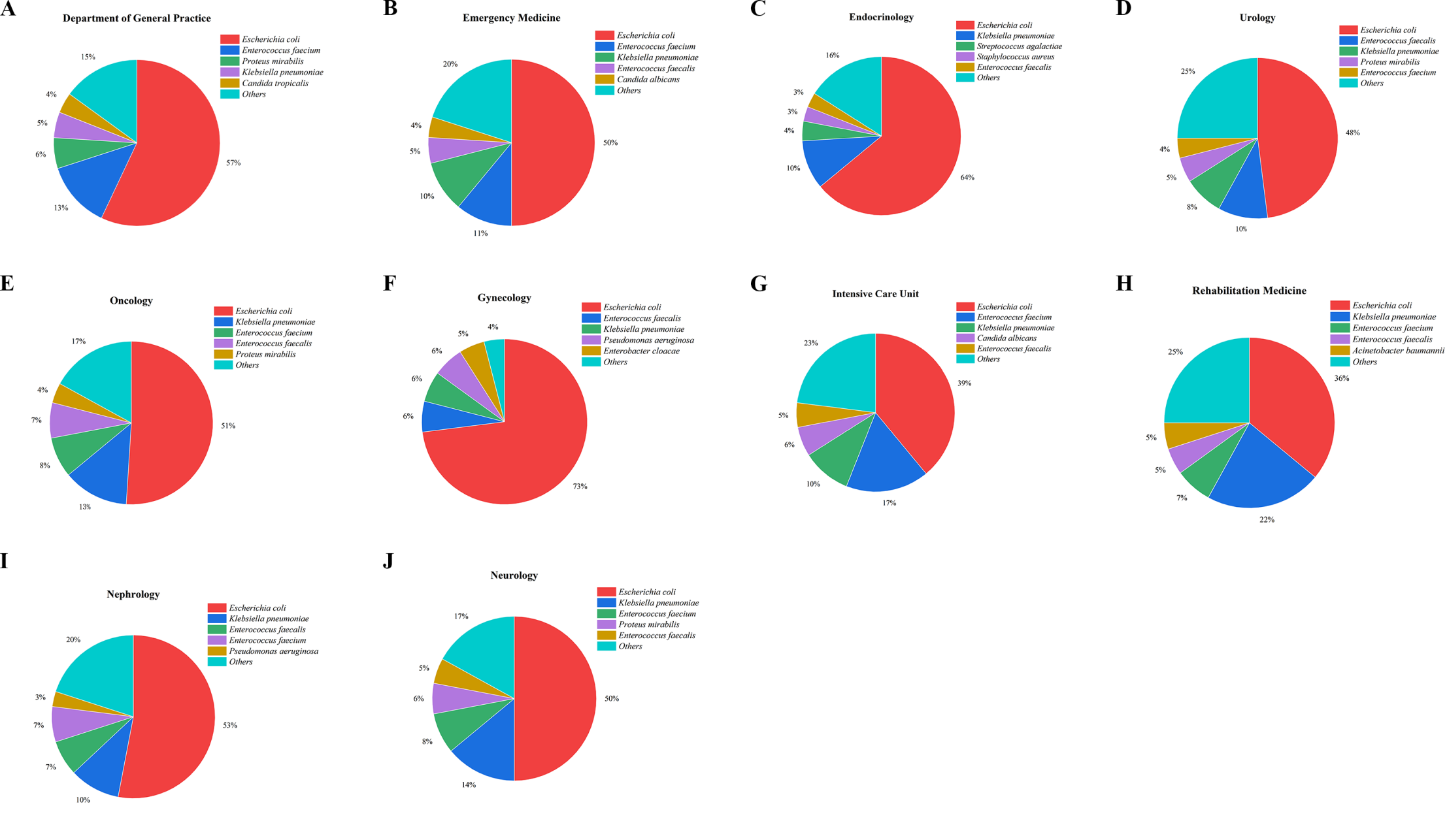
**

Figure S2 Distribution of uropathogens across different clinical departments among patients with urinary tract infection.

Table S1 Antimicrobial Susceptibility Characteristics of Major Gram-Negative Urinary Tract Bacteria in Different Patient Groups

| **Pathogen** | **Antibiotic agent** | **Age (years)** | | | | **Gender** | | |
| --- | --- | --- | --- | --- | --- | --- | --- | --- |
|  |  | **18-44 (n = 233)** | **45-64 (n = 713)** | **≥65 (n = 628)** | ***p*** | **Male (n = 409)** | **Female (n = 1165)** | ***p*** |
| ***E.coli***  **(n =1,574)** | ESBL | 47.4 | 49.2 | 48.9 | 0.865 | 55.8 | 46.4 | 0.001 |
|  | AMC | 12.5 | 9.9 | 10.6 | 0.563 | 15.3 | 8.9 | <0.001 |
|  | CSL | 6.8 | 3.4 | 6.5 | 0.014 | 7.8 | 4.2 | 0.004 |
|  | TZP | 5.6 | 4.8 | 6.2 | 0.509 | 8.4 | 4.5 | 0.003 |
|  | CXM | 52.6 | 53.7 | 53.4 | 0.969 | 61.9 | 50.5 | <0.001 |
|  | CAZ | 26 | 22 | 22.9 | 0.423 | 30.4 | 20.4 | <0.001 |
|  | CRO | 50.9 | 52.4 | 51.5 | 0.903 | 60.6 | 48.8 | <0.001 |
|  | FEP | 12.5 | 8.1 | 13.3 | 0.006 | 16.5 | 8.8 | <0.001 |
|  | FOX | 15.1 | 10.8 | 11.8 | 0.223 | 16.1 | 10.3 | 0.002 |
|  | ETP | 3 | 1.6 | 2.6 | 0.284 | 3.8 | 1.6 | 0.007 |
|  | IPM | 2.1 | 1.3 | 1.9 | 0.524 | 2.4 | 1.4 | 0.144 |
|  | AMK | 2.1 | 2.8 | 2.2 | 0.748 | 3.2 | 2.2 | 0.289 |
|  | LEV | 43.2 | 52.9 | 57.7 | <0.001 | 54.5 | 52.9 | 0.566 |
|  | SXT | 56.8 | 46.4 | 47.4 | 0.021 | 48.8 | 48.3 | 0.842 |
| ***K.pneumoniae***  **(n = 300)** |  | **18-44 (n = 33)** | **45-64 (n = 142)** | **≥65 (n = 125)** | ***p*** | **Male (n = 112)** | **Female (n = 188)** | ***p*** |
|  | ESBL | 32.3 | 31.7 | 43.5 | 0.137 | 36.7 | 36.7 | 0.987 |
|  | AMC | 31.2 | 24.1 | 24.2 | 0.728 | 30.3 | 21.8 | 0.098 |
|  | CSL | 18.2 | 16.9 | 13.6 | 0.696 | 23.2 | 11.2 | 0.006 |
|  | TZP | 18.8 | 20 | 22.6 | 0.806 | 30.6 | 15.1 | 0.001 |
|  | CXM | 48.5 | 48.6 | 55.2 | 0.527 | 58 | 47.3 | 0.073 |
|  | CAZ | 25 | 35.9 | 38.7 | 0.319 | 46.4 | 29.8 | 0.004 |
|  | CRO | 42.4 | 45.8 | 52 | 0.474 | 56.2 | 43.1 | 0.027 |
|  | FEP | 31.2 | 35 | 40.3 | 0.521 | 43.2 | 33 | 0.086 |
|  | FOX | 21.9 | 21.8 | 16.9 | 0.571 | 25.5 | 16.5 | 0.049 |
|  | ETP | 12.5 | 13.4 | 9.7 | 0.624 | 20 | 6.9 | <0.001 |
|  | IPM | 9.1 | 12 | 8.8 | 0.676 | 17.9 | 5.9 | <0.001 |
|  | AMK | 9.1 | 10.6 | 12.8 | 0.771 | 17 | 8 | 0.018 |
|  | LEV | 36.4 | 34.5 | 40.8 | 0.565 | 45.5 | 32.4 | 0.023 |
|  | SXT | 42.4 | 38.7 | 49.6 | 0.200 | 52.7 | 38.3 | 0.015 |
|  |  | **18-44 (n = 23)** | **45-64 (n = 51)** | **≥65 (n = 55)** | ***p*** | **Male (n = 39)** | **Female (n =90)** | ***p*** |
| ***P.mirabilis***  ***(n = 129)*** | AMC | 21.7 | 27.5 | 29.1 | 0.799 | 33.3 | 24.7 | 0.297 |
|  | CSL | 0 | 0 | 0 |  | 0 | 0 |  |
|  | TZP | 0 | 0 | 0 |  | 0 | 0 |  |
|  | CXM | 30.4 | 39.2 | 46.4 | 0.364 | 56.4 | 33.3 | 0.014 |
|  | CAZ | 4.3 | 2 | 1.8 | 0.807 | 5.1 | 1.1 | 0.188 |
|  | CRO | 30.4 | 27.5 | 39.3 | 0.371 | 43.6 | 27.8 | 0.078 |
|  | FEP | 0 | 0 | 0 |  | 0 | 0 |  |
|  | FOX | 0 | 7.8 | 5.5 | 0.214 | 5.1 | 5.6 | 0.921 |
|  | ETP | 0 | 0 | 0 |  | 0 | 0 |  |
|  | IPM | 15 | 36.7 | 29.4 | 0.086 | 31.6 | 29.6 | 0.930 |
|  | AMK | 0 | 0 | 1.8 | 0.424 | 0 | 1.1 | 0.395 |
|  | LEV | 34.8 | 37.3 | 37.5 | 0.961 | 41 | 34.4 | 0.476 |
|  | SXT | 56.5 | 54.9 | 75 | 0.082 | 66.7 | 62.2 | 0.630 |

Notes: AMC, amoxicillin–clavulanate; CSL,cefoperazone–sulbactam; TZP, piperacillin–tazobactam; CXM, cefuroxime; CAZ, ceftazidime; CRO, ceftriaxone; FEP, cefepime; FOX, cefoxitin; ETP, ertapenem; IPM, imipenem; AMK ,amikacin; LEV, levofloxacin; SXT, trimet hoprim–sulfamethoxazole; ESBL, Extended-Spectrum Beta-Lactamase.

Table S2 Antimicrobial Susceptibility Characteristics of Major Gram-Positive Urinary Tract Bacteria in Different Patient Groups

| **Pathogen** | **Antibiotic agent** | **Age (years)** | | | | **Gender** | | |
| --- | --- | --- | --- | --- | --- | --- | --- | --- |
|  |  | **18-44(n = 47)** | **45-64(n = 79)** | **≥65(n = 113)** | ***p*** | **Male(n = 146)** | **Female (n = 93)** | ***p*** |
| ***E. faecalis***  **(n= 239)** | PEN | 0 | 1.3 | 1.8 | 0.501 | 2.1 | 0 | 0.084 |
|  | AMP | 0 | 0 | 0 |  | 0 | 0 |  |
|  | GEH | 29.8 | 32.4 | 36.7 | 0.691 | 36.7 | 29.7 | 0.275 |
|  | LEV | 19.1 | 29.1 | 31 | 0.289 | 25.3 | 32.3 | 0.246 |
|  | LNZ | 4.3 | 7.7 | 2.7 | 0.273 | 3.5 | 6.5 | 0.276 |
|  | VAN | 0 | 0 | 0 |  | 0 | 0 |  |
| ***E. faecium***  **(n= 220)** |  | **18-44**  **(n = 19)** | **45-64**  **(n = 72)** | **≥65**  **( n = 129)** | ***p*** | **Male**  **(n = 102)** | **Female**  **(n = 118)** | ***p*** |
|  | PEN | 94.7 | 100 | 100 | 0.084 | 99 | 100 | 0.214 |
|  | AMP | 89.5 | 98.6 | 99.2 | 0.084 | 97.1 | 99.2 | 0.239 |
|  | GEH | 44.4 | 29 | 37 | 0.408 | 37.1 | 33.3 | 0.514 |
|  | LEV | 89.5 | 98.6 | 99.2 | 0.084 | 97.1 | 99.2 | 0.239 |
|  | LNZ | 0 | 1.4 | 0.8 | 0.766 | 0 | 1.7 | 0.113 |
|  | VAN | 0 | 5.6 | 3.1 | 0.337 | 2.9 | 4.2 | 0.606 |

**Notes: PEN, penicillin; AMP, ampicillin; GEH, high-level gentamicin; LNZ, linezolid; VAN, vancomycin; LEV, levofloxacin.**

Table S3 Department‑stratified antimicrobial resistance rates of major uropathogens, 2021–2024

| ****Antibiotics**** | **Urology** | | | **Nephrology** | | | **Emergency Department** | | | **Rehabilitation Department** | | |
| --- | --- | --- | --- | --- | --- | --- | --- | --- | --- | --- | --- | --- |
|  | **E. Coli**  **(724)** | **E.faecalis**  **(149)** | **K.pneumoniae**  **(114)** | **E.Coli**  **(124)** | **K.pneumoniae**  **(23)** | **E.faecalis**  **(17)** | **E.Coli**  **(106)** | **E.faecium**  **(23)** | **K.pneumoniae**  **(22)** | **E.Coli**  **(53)** | **K.pneumoniae**  **(32)** | **E.faecium**  **(10)** |
| ESBL | 52 |  | 35.7 | 35.8 | 43.5 |  | 50 |  | 42.9 | 54.9 | 40.6 |  |
| AMC | 10.1 |  | 18 | 9.8 | 4.3 |  | 9.5 |  | 27.3 | 15.7 | 43.8 |  |
| CSL | 4.8 |  | 7 | 5.6 | 4.3 |  | 8.5 |  | 22.7 | 5.7 | 34.4 |  |
| TZP | 5 |  | 12.6 | 4.9 | 9.1 |  | 5.7 |  | 27.3 | 7.5 | 34.4 |  |
| CXM | 56.2 |  | 44.7 | 41.9 | 43.5 |  | 53.8 |  | 63.6 | 62.3 | 75 |  |
| CAZ | 24.8 |  | 22.3 | 11.5 | 26.1 |  | 23.1 |  | 45.5 | 27.5 | 65.6 |  |
| CRO | 54.1 |  | 40.4 | 38.7 | 43.5 |  | 50.9 |  | 63.6 | 60.4 | 68.8 |  |
| FEP | 12.1 |  | 24.3 | 5.7 | 22.7 |  | 8.6 |  | 54.5 | 13.5 | 59.4 |  |
| FOX | 13 |  | 11.6 | 9.8 | 0 |  | 15.2 |  | 22.7 | 13.7 | 43.8 |  |
| ETP | 2.1 |  | 2.7 | 0 | 0 |  | 3.8 |  | 18.2 | 5.9 | 28.1 |  |
| IPM | 1.2 |  | 2.6 | 0 | 0 |  | 2.8 |  | 18.2 | 5.7 | 28.1 |  |
| AMK | 3 |  | 6.1 | 2.4 | 0 |  | 1.9 |  | 27.3 | 3.8 | 25 |  |
| LEV | 55.2 | 26.2 | 33.3 | 49.2 | 34.8 | 11.8 | 52.8 | 100 | 50 | 58.5 | 59.4 | 100 |
| SXT | 47.3 |  | 38.6 | 53.2 | 39.1 |  | 51.9 |  | 59.1 | 62.3 | 56.2 |  |
| PEN |  | 0.7 |  |  |  | 0 |  | 100 |  |  |  | 100 |
| AMP |  | 0 |  |  |  | 0 |  | 100 |  |  |  | 100 |
| GEH |  | 34 |  |  |  | 20 |  | 43.5 |  |  |  | 40 |
| LNZ |  | 5.4 |  |  |  | 0 |  | 0 |  |  |  | 0 |
| VAN |  | 0 |  |  |  | 0 |  | 8.7 |  |  |  | 0 |

Notes: AMC, amoxicillin–clavulanate; CSL,cefoperazone–sulbactam; TZP, piperacillin–tazobactam; CXM, cefuroxime; CAZ, ceftazidime; CRO, ceftriaxone; FEP, cefepime; FOX, cefoxitin; ETP, ertapenem; IPM, imipenem; AMK ,amikacin; LEV, levofloxacin; SXT, trimet hoprim–sulfamethoxazole; ESBL, Extended-Spectrum Beta-Lactamase; **PEN, penicillin; AMP, ampicillin; GEH, high-level gentamicin; LNZ, linezolid; VAN, vancomycin.**

Table S4 Department‑stratified antimicrobial resistance rates of major uropathogens, 2021–2024

| ****Antibiotics**** | **Endocrinology** | | **Oncology** | | **Gynecology** | | **General Practice** | | **Neurology** | | **Intensive Care Unit** | |  |
| --- | --- | --- | --- | --- | --- | --- | --- | --- | --- | --- | --- | --- | --- |
|  | **E.Coli**  **(87)** | **K.pneumoniae**  **(13)** | **E.Coli**  **(57)** | **K.pneumoniae**  **(14)** | **E.Coli**  **(64)** | **E.faecalis**  **(5)** | **E.Coli**  **(45)** | **E.faecium**  **(10)** | **E.Coli**  **(39)** | **K.pneumoniae**  **(11)** | **E. Coli(98)** | **E. Faecium(42)** | **K.pneumoniae(26)** |
| ESBL | 33.7 | 7.7 | 59.6 | 57.1 | 67.2 |  | 37.8 |  | 43.6 | 45.5 | 52 |  | 23.1 |
| AMC | 4.6 | 0 | 12.3 | 14.3 | 9.7 |  | 13.3 |  | 12.8 | 54.5 | 9.2 |  | 42.3 |
| CSL | 1.1 | 0 | 7 | 0 | 6.2 |  | 4.4 |  | 7.7 | 54.5 | 7.1 |  | 34.6 |
| TZP | 6.9 | 0 | 8.8 | 14.3 | 1.6 |  | 6.7 |  | 10.3 | 54.5 | 7.1 |  | 30.8 |
| CXM | 35.6 | 7.7 | 63.2 | 57.1 | 65.6 |  | 42.2 |  | 51.3 | 72.7 | 55.1 |  | 53.8 |
| CAZ | 13.8 | 7.7 | 22.8 | 57.1 | 27.4 |  | 26.7 |  | 25.6 | 54.5 | 25 |  | 46.2 |
| CRO | 34.5 | 7.7 | 59.6 | 57.1 | 69.8 |  | 42.2 |  | 51.3 | 72.7 | 52 |  | 50 |
| FEP | 6.9 | 7.7 | 15.8 | 50 | 7.8 |  | 13.3 |  | 12.8 | 72.7 | 18.4 |  | 50 |
| FOX | 4.6 | 0 | 14 | 7.1 | 12.9 |  | 11.1 |  | 7.7 | 45.5 | 5.1 |  | 34.6 |
| ETP | 1.1 | 0 | 3.5 | 0 | 1.6 |  | 0 |  | 5.1 | 27.3 | 1 |  | 30.8 |
| IPM | 1.1 | 0 | 3.5 | 0 | 1.6 |  | 0 |  | 5.1 | 27.3 | 1 |  | 30.8 |
| AMK | 0 | 0 | 3.5 | 7.1 | 3.1 |  | 2.2 |  | 0 | 27.3 | 1 |  | 19.2 |
| LEV | 52.9 | 7.7 | 57.9 | 57.1 | 48.4 | 80 | 57.8 | 100 | 53.8 | 54.5 | 46.9 | 100 | 23.1 |
| SXT | 37.9 | 15.4 | 57.9 | 64.3 | 59.4 |  | 48.9 |  | 46.2 | 45.5 | 48.5 |  | 42.3 |
| PEN |  |  |  |  |  | 0 |  | 100 |  |  |  | 100 |  |
| AMP |  |  |  |  |  | 0 |  | 100 |  |  |  | 97.6 |  |
| GEH |  |  |  |  |  | 20 |  | 20 |  |  |  | 35.7 |  |
| LNZ |  |  |  |  |  | 0 |  | 0 |  |  |  | 2.4 |  |
| VAN |  |  |  |  |  | 0 |  | 0 |  |  |  | 2.4 |  |

Notes: AMC, amoxicillin–clavulanate; CSL,cefoperazone–sulbactam; TZP, piperacillin–tazobactam; CXM, cefuroxime; CAZ, ceftazidime; CRO, ceftriaxone; FEP, cefepime; FOX, cefoxitin; ETP, ertapenem; IPM, imipenem; AMK ,amikacin; LEV, levofloxacin; SXT, trimet hoprim–sulfamethoxazole; ESBL, Extended-Spectrum Beta-Lactamase; **PEN, penicillin; AMP, ampicillin; GEH, high-level gentamicin; LNZ, linezolid; VAN, vancomycin.**
